# Orthology and synteny analysis of receptor-like kinases “RLK” and receptor-like proteins “RLP” in legumes

**DOI:** 10.1186/s12864-021-07384-w

**Published:** 2021-02-10

**Authors:** Daniel Restrepo-Montoya, Phillip E. McClean, Juan M. Osorno

**Affiliations:** 1 Genomics and Bioinformatics Program, North Dakota State University, Fargo, ND 58108-6050 USA; 2 Department of Plant Sciences, North Dakota State University, Fargo, ND 58108-6050 USA

**Keywords:** Dicots, Plasma membrane receptors, Target synteny blocks, Legumes/non-legumes

## Abstract

**Background:**

Legume species are an important plant model because of their protein-rich physiology. The adaptability and productivity of legumes are limited by major biotic and abiotic stresses. Responses to these stresses directly involve plasma membrane receptor proteins known as receptor-like kinases and receptor-like proteins. Evaluating the homology relations among RLK and RLP for seven legume species, and exploring their presence among synteny blocks allow an increased understanding of evolutionary relations, physical position, and chromosomal distribution in related species and their shared roles in stress responses.

**Results:**

Typically, a high proportion of RLK and RLP legume proteins belong to orthologous clusters, which is confirmed in this study, where between 66 to 90% of the RLKs and RLPs per legume species were classified in orthologous clusters. One-third of the evaluated syntenic blocks had shared RLK/RLP genes among both legumes and non-legumes. Among the legumes, between 75 and 98% of the RLK/RLP were present in syntenic blocks. The distribution of chromosomal segments between *Phaseolus vulgaris* and *Vigna unguiculata*, two species that diverged ~ 8 mya, were highly similar. Among the RLK/RLP synteny clusters, seven experimentally validated resistance RLK/RLP genes were identified in syntenic blocks. The RLK resistant genes FLS2, BIR2, ERECTA, IOS1, and AtSERK1 from *Arabidopsis* and SLSERK1 from *Solanum lycopersicum* were present in different pairwise syntenic blocks among the legume species. Meanwhile, only the LYM1- RLP resistant gene from *Arabidopsis* shared a syntenic blocks with *Glycine max*.

**Conclusions:**

The orthology analysis of the RLK and RLP suggests a dynamic evolution in the legume family, with between 66 to 85% of RLK and 83 to 88% of RLP belonging to orthologous clusters among the species evaluated. In fact, for the 10-species comparison, a lower number of singleton proteins were reported among RLP compared to RLK, suggesting that RLP positions are more physically conserved compared to RLK. The identification of RLK and RLP genes among the synteny blocks in legumes revealed multiple highly conserved syntenic blocks on multiple chromosomes. Additionally, the analysis suggests that *P. vulgaris* is an appropriate anchor species for comparative genomics among legumes.

**Supplementary Information:**

The online version contains supplementary material available at 10.1186/s12864-021-07384-w.

## Background

Legumes are derived from a common ancestor 60 million years ago (mya) [1]. Based on morphological characters, three major legume subfamilies exist: mimosoids (Mimosoideae), caesalpiniods (Caesalpinioideae), and papilionoids (Papilionoideae). The latter subfamily contains the cultivated grain legumes or pulses and can be subdivided into four clades: 1) Phaseoloids: *Glycine* spp. Willd., *Phaseolus* spp. L., *Cajanus* spp. L., and *Vigna* spp. Savi; 2) Galeogoids: *Pisum* L., *Lens* Mill., *Lathyrus* L., *Vicia* L., *Medicago* L., and *Cicer* L.; 3) Genistoids: *Lupinus* L.; and 4) Dalbergoids: *Arachis* L. [2]. In most cases, the domestication of the Fabaceae (Syn. Leguminosae) family as grain legumes has been reported in conjunction with cereals [3]. However, more legumes have been domesticated overall, which makes the Fabaceae family the taxon with the greater number of domesticates [3, 4]. Of the legume clades, the Phaseolid group of warm-season legumes was domesticated later than the Galeogoids group of cool-season legumes [4].

The Papilionoideae subfamily, the largest clade among the legumes, is monophyletic. It shares a common ancestor, and its chloroplast experienced a 50 kb inversion 50 mya [1]. Research shows that the timing of polyploidy (whole genome duplication, or WGD), which affects most lineages in this clade, occurred after the divergence of the mimosoid and papilionoid clades, but the precise timing is still unknown [5]. Among the most recognized legumes significant genomic resources available are *Medicago truncatula* L [6], pigeon pea (*Cajanus cajan* L.) [7], soybean (*G. max* (L.) Merrill), mungbean (*Vigna radiata* (L.) R. Wilczek) [8], cowpea (*V. unguiculata* L. Walp) [9], adzuki bean (*Vigna angularis* var. *angularis*) [10], and common bean (*P. vulgaris* L.) [11]. In 2005, WGD events were reported that established the legume phylogenetic relationship [1]. Interestingly, during the last 135 to 250 million years of evolution, the protein-coding gene families have been affected by different biological events, such as various gene duplication mechanisms, including WGDs (or polyploidization) as well as segmental and tandem duplications, among other processes [12–14].

**Fig. 1:**
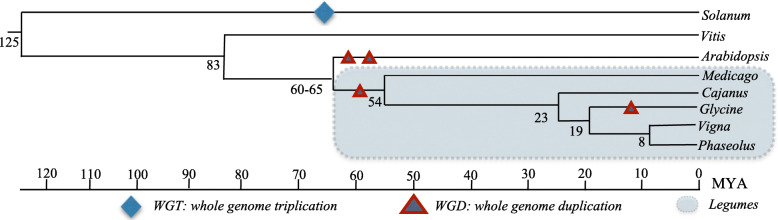
Taxonomic relationships among legumes/non-legumes. The topology and distances reported were adapted from [1, 17, 18]. *S. lycopersicum*, *V. vinifera* and *Arabidopsis thaliana* were included as outgroup species for this study

In legumes, several WGD and triplication events occurred soon after the monocots and eudicots split evolutionarily [15]. Common grape (*Vitis vinifera* L.) divergence is known to have occurred early in eudicot evolution; due to this event, grape is considered ideal for studies of chromosomal evolution among dicots [15]. Based on the fossil records, the divergence of Fabales from the Rosales and Cucurbitales was estimated at 59.9 mya. A Papilionoideae-specific WGD was observed among legumes [5], and recent duplications occurred in soybean about 13 mya [16]. Soybean, pigeonpea, mungbean, and common bean evolved from a common ancestor about 23.9 mya (Fig. 1).

The release of reference genome sequences of legumes [17] enables comparative genomic analyses. Such research requires a complex genome annotation process that depends on identifying homologous sequences as orthologs to sequences of known identity and function. Orthologous genes (orthologs) are the result of speciation events that are derived from a common ancestor [19] and are predicted to have conserved all or part of an ancestral biological function [20]. Comparative genome analyses can identify ortholog clusters, single-copy genes, and singletons that are conserved through evolutionary time [21] and are not present in any orthologous group or remain ungrouped [22]. This sort of analysis is ideal for RLK, RLP, and RLCKs (cytoplasmic RLK) because of their evolutionary relationships, their important roles in plant signaling, and because their gene subfamilies are large with complex histories of gene duplication and loss [23]. The evaluation of RLK/RLP among *Aradidopsis*, *Lotus japonica,* and *M. truncatula* discovered gene duplication and a high frequency of reciprocal gene loss in the LRR-RLK/RLP, and RLCK subfamilies. Furthermore, pairwise comparisons showed lineage-specific duplications associated with reciprocal gene loss [23].

Extensive genetic and phenotypic studies have reported diverse functional roles of RLK and RLP (plasma membrane receptors) extending from the control of cell development to stress responses [24]. These receptors play a crucial role in plant disease resistance [25]. According to the innate immunity plant system described by the zigzag model [26], the RLK and RLP are considered the first line of plant cell defense for some host-pathogen interactions, which can be a constituent of both non-host and host resistance [26–28]. RLK proteins are structurally similar to RLP, but the RLP does not have a cytoplasmic kinase domain [29]. Also, the plasma membrane receptors present a diverse set of extracellular domains such as the leucine-rich repeat “LRR” [30], different domains related to the lectin family [31], or the cell-wall associated kinase “WAK” [32], among other domains. The structural details of plasma membrane receptors have been described by different authors [32–35]. The RLK/RLP identification and comparative genomic evaluation, like synteny analysis, could lead to the development of high-density receptor candidates for genetic maps and crop improvement [36].

Synteny analysis is a useful strategy to investigate evolutionary relationships and to identify functionally related genes [37]. Syntenic blocks are defined as groups of genes that exhibit conserved gene order across genomes [38], and the blocks are identified by homology analysis across genomes. For synteny analysis, the focus is on homologous genes classified as orthologs based on speciation events [39]. Structural homologies can be evaluated at the micro- or macrosynteny level. Microsynteny analysis evaluates narrow regions of the genome, while macrosynteny analysis focuses on chromosomal or whole genome comparisons [40]. Recently, synteny comparisons between closely-related eukaryotic species determined that homologous genes remained on corresponding chromosomes [12]. Today, a common strategy to infer function from homology is directly related to ortholog identification [41]. Most tools used today to define synteny consider homology as a matter of principle and orthology as a result of practical constraints [38].

One aspect of genome-wide comparative genomics is to identify genomic segments of conserved orthologous gene order at the chromosomal level among species at different levels of evolutionary relatedness [42]. This allows an understanding of evolutionary processes that lead to a diversity of chromosome number and structural lineages across multiple species. Interestingly, many tools use orthologous relationships between protein-coding genes as anchors to position statistically significant local alignments [43]. The identification of syntenic regions containing RLK/RLP receptors between non-legume and legume species is an efficient strategy to identify patterns of evolutionary conservation and divergence across genomes for a class of proteins involved in many aspects of plant growth, development, and response to biotic and abiotic stresses [44].

Among legumes, it has been reported that macrosynteny in species such as *M. truncatula* and *G. max* can be as long as the chromosome arms or span most of the euchromatin region of the two genomes*.* Each *M. truncatula* region and its homeologue typically show similarity to three *V. vinifera* regions via the pre-rosid whole genome hexaploidy [45]. Within the millettioid clade, pigeonpea (*C. cajan*) diverged from the soybean ~ 20–30 mya. Interestingly, after this long period of divergence, high levels of synteny are still observed between these two species [7]. Each pigeonpea chromosome shows extensive synteny with two or more soybean chromosomes, likely due to an independent soybean duplication event [16]. Also, the genome comparison of *V. radiata* var. *radiata* with *A. thaliana*, *Cicer arietinum*, *C. cajan*, *G. max*, *L. japonicas,* and *M. truncatula* revealed well-conserved macrosynteny blocks, although these blocks were highly dispersed among plant species with different numbers of chromosomes [8].

To understand the structural relationships between the common bean and soybean genome, syntenic gene-rich regions were identified for all soybean chromosomes in precise regions of the common bean genetic map [37]. The research concluded that, relative to common bean, soybean is segmentally rearranged, exhibiting evidence of a one-to-two relationship, respectively [37]. Among the *Vigna* genus, cowpea (*V. unguiculata*) shares a high degree of collinearity with *P. vulgaris* [46]. Muñoz-Amatriaín et al. in 2017 explored the genetic diversity along each linkage group among *V. unguiculata* and *P. vulgaris* and found the groups to have macrosynteny [47]. In contrast, given the close relationship of *Vigna* to *Glycine*, most of the *V. radiata* var. *radiata* genes were found in synteny to *G. max*. Of the 18,378 *V. radiata* genes on pseudo-chromosomes, 14,569 were located in 1059 synteny blocks of orthologues or paralogues with soybean [8]. It was also reported that 11,853 mungbean genes were in synteny with the *C. cajan* genome [8].

Based on a previous computational identification of RLK/RLP in legume species [48], an orthology and synteny analysis of the plasma membrane receptors were undertaken to describe the physical relationship of RLK- and RLP proteins among legumes/non-legumes. The seven legumes involved in this evaluation were *G. max* GM, *P. vulgaris* PV, *M. truncatula* MT, *V. angularis* VA, *V. radiata* VR, *V. unguiculata* VU, and *C. cajan* CC. Three non-legume species were used as the outgroup species: *A. thaliana* (L.,) Heynh [49], tomato (*S. lycopersicum* (L.) H. Karst) SL [50], and common grape (*V. vinifera* L.) VV [51]. The first two species were included because many RLK/RLP proteins related to them have been experimentally-validated [48]. Grape represents the basal rosid lineage and has close-to-ancestral karyotypes that facilitate comparisons across major eurosids [13, 51]. The purpose of this analysis was 1) to establish the RLK/RLP homology relationship among legumes and 2) to evaluate the distribution, conservation, and divergence of the pairwise RLK/RLP syntenic blocks. It also used the experimentally-validated RLK/RLP resistance genes [48] to target synteny blocks. The analysis evaluated the chromosomal segment distribution of syntenic blocks with RLK/RLP among the species to identify patterns of evolutionary conservation and divergence. This information was also used independently with *P. vulgaris* and *V. vinifera* chromosomes as a reference model for the comparison of RLK/RLP synteny blocks among the legume and non-legume species to illustrate genomic structural divergence, due to the fact that not many studies in legume species have been yet dedicated to RLK and RLP protein analysis [52, 53].

## Results

### Orthology analysis of RLK-RD

**Fig. 2:**
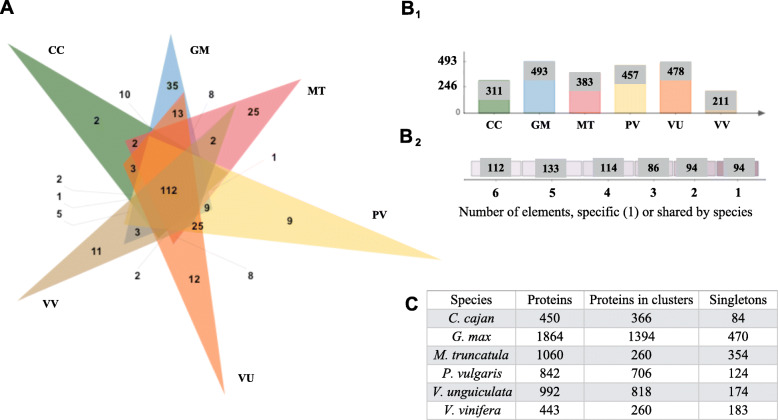
Summary of the RLK-RD orthology analysis. **A**. Venn diagram showing the distribution of shared RLK-RD gene families (orthologous clusters) among CC, GM, PV, MT, VU, and VV. **B1**. The numbers refer to all the clusters in the species, including orthologs and in-paralogs **B2**. Distribution of the number of species present in orthologous clusters with one or more shared elements among species. **C**. Summary of the total number of proteins, clusters, and singletons within each species. The RLK and its isoforms and non-RD proteins were included in this analysis

The five-legume species CC, GM, PV, MT, VU, and VV as the outgroup, were selected for the orthology analyses. *V. unguiculata* (VU) was selected as the *Vigna* sp. representative because of the quality of its reference genome assembly and annotation [54]. Data for the remaining species, VR, VA, SL, and AT, were included in the supplementary material. The orthology and hierarchical clustering domain analyses of RLK for the legume species resulted in the formation of 633 orthologous and paralogous clusters (RLK proteins in clusters related to one until six species Fig. 2: B2), 539 orthologous clusters containing at least two species (RLK proteins in clusters Fig. 2: B2), and seven single-copy gene clusters (Additional file 1: Table S1). In total, 112 orthologous clusters contained all six species and the outgroup. Also, clusters unique to each of the six species, presumably formed by within species duplication, were identified (Fig. 2: A and B2). The remaining 427 orthologous clusters were shared by at least two legume species, with 87 orthologous clusters shared by all five-legume species. *G. max* was the species with the most singletons (Fig. 2: C) and proteins present in orthologous clusters (Fig. 2: B1). 462 orthologous clusters for VR, VA, AT, VV, and SL were identified. 411 out of the 462 clusters contain proteins from at least two species. In particular, 107 clusters contained proteins from five species, and 28 single-copy gene clusters were reported (Additional file 2: Figure S1, Additional file 3: Table S2). The RLK-nonRD were also included in the analysis to evaluate the whole set of RLK proteins (Fig. 2).

### Orthology of RLK-nonRD

**Fig. 3:**
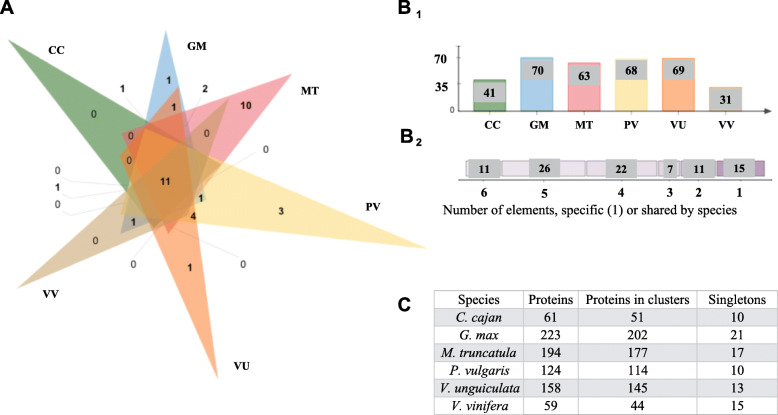
Summary of the RLK-nonRD orthology analysis. **A**. Venn diagram showing the distribution of shared RLK-nonRD gene families (orthologous clusters) among CC: *C. cajan*, GM: *G. max*, PV: *P. vulgaris*, VV: *V. vinifera* “outgroup,” VU: *V. unguiculata*, and MT: *M. truncatula*. **B1**. The numbers refer to all the clusters in the species, including orthologs and in-paralogs. **B2**. Distribution of the number of species present in orthologous clusters, elements 1, or shared among species lists. **C.** Summary of the total number of proteins, clusters, and singletons within each species. The RLK-nonRD isoforms are included in this analysis

Results for the RLK-nonRD were included in the RLK orthology analysis (Fig. 2), to determine their distribution among CC, GM, PV, MT, VU, and VV; the RLK-nonRD are shown individually in Fig. 3. The RLK-nonRD proteins formed 92 orthologous and paralogous clusters, 77 orthologous clusters contained at least two species, and two single-copy clusters (Additional file 4: Table S3). In total, 11 orthologous clusters identified were shared by all five species and the outgroup. PV, GM, MT, and VU showed unique orthologous clusters. Notably, MT-specific clusters were diverse compare to the other 5 species, (Fig. 3: A). Of those remaining clusters, 66 were shared by at least two legume species, and 13 were shared by all five legumes species (Fig. 3: B2). *G. max* had the most singletons (Fig. 3: C) and proteins present in orthologous clusters (Fig. 3: B1). The unique clusters were formed by paralogous or protein isoforms belonging to the same gene (Fig. 3: A and B2). The orthology results for VR, VA, AT, VV, and SL formed 71 clusters, and 65 of the orthologous clusters contained a minimum of two species. Specifically, 12 orthologous clusters had proteins from five species, and three single-copy gene clusters were reported (Additional file 5: Figure S2, Additional file 6: Table S4).

### Orthology analysis of RLP

**Fig. 4:**
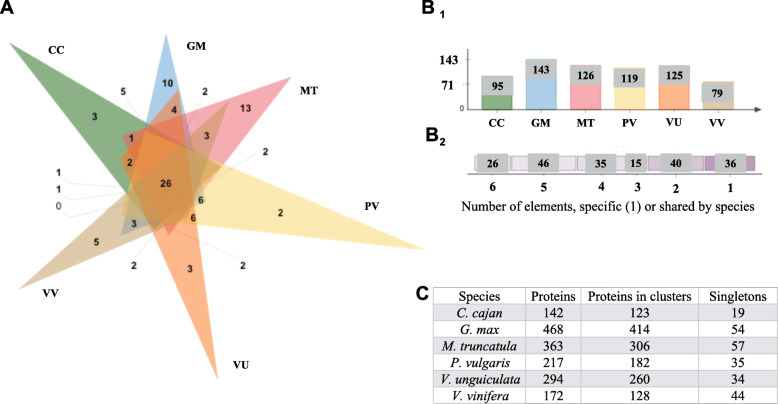
Summary of the RLP orthology analysis. **A**. Venn diagram showing the distribution of shared RLP gene families (orthologous clusters) among CC, GM, PV, MT, VU, and VV. **B1**. The numbers refer to all the clusters in the species, including orthologs and in-paralogs. **B2**. Distribution of the number of species present in orthologous clusters, elements 1, or shared among species. **C**. Summary of the total number of proteins, clusters, and singletons within each species

The orthology analysis of RLP for the five-legume species CC, GM, PV, MT, VU, and VV as outgroup identified 198 orthologous and paralogous clusters, 162 orthologous clusters containing at least two species, and one single-copy gene cluster for each species (Additional file 7: Table S5). In total, 26 orthologous clusters were identified among the six-species analysis that included the outgroup. All species showed unique clusters (Fig. 4: A and B2). The remaining 136 orthologous clusters were shared by at least two legume species, and 21 orthologous clusters were shared by all five-legume species. *M. truncatula* was the species with the most singletons overall (Fig. 4: C), with *G. max* as the species with the most proteins present in orthologous clusters (Fig. 4: B1). Unique clusters were formed by paralogous or protein isoforms belonging to the same gene (Fig. 4: A and B2). The orthology analysis for VR, VA, AT, VV and SL formed143 orthologous clusters, and 122 of these contained at least two species, 25 orthologous clusters were represented by proteins from five species, and 4 single-copy gene clusters were reported (Additional file 8: Figure S3 and Additional file 9: Table S6).

### Synteny analysis

The syntenic block analysis identified 690,397 matches, 6252 pairwise comparisons, 9011 alignments or pairwise clusters, and 3592 alignments with RLK/RLP proteins. These represent the whole set of synteny blocks shared among the legumes and non-legumes species. The whole syntenic block set was split using the RLK/RLP genes as a reference to identify the sets of synteny blocks with the presence of plasma membrane receptors. Among all the genes initially processed for the species evaluated, 70 and 82% of the total RLK and RLP, respectively, were physically identified in chromosomes. 77 and 72% of the RLK/RLP, respectively, were located in 1 or more synteny blocks (Additional file 10: Table S7).

The presence and distribution of RLK/RLP genes in the interspecies synteny blocks and the identification of the plasma membrane receptors and their general distribution among the species are shown in Table 1. In most cases, the number of legume/non-legume genes belonging to one or more synteny blocks per species was higher compared with those genes that do not belong to the blocks. The exceptions are the AT RLK genes, the AT and SL RLK-nonRD genes, and the VV, AT, and SL RLP genes. All legumes (CC, GM, MT, PV, VA, VU, and VR) showed a higher proportion of RLK/RLP genes located in synteny blocks. All RLK-nonRD genes present in the PV and VU genomes were in synteny blocks, and among legumes, the MT species had the fewest RLK/RLPs proteins present in blocks (Table 1).

**Table 1:** Summary of RLK and RLP genes among species in synteny blocks

Species	RLK no blocks	RLK in blocks^a^	Freq. range	RLK-nonRD no blocks	RLK-nonRD in blocks^a^	Freq. range	RLP no blocks	RLP in blocks^a^	Freq. range
CC	38	166	1 to 9	8	21	1 to 7	16	50	1 to 8
GM	57	907	1 to 13	17	148	1 to 9	54	336	1 to 9
MT	116	525	1 to 9	88	95	1 to 9	154	164	1 to 7
PV	7	520	1 to 10	0	113	1 to 8	6	193	1 to 8
VA	8	427	1 to 9	3	78	1 to 7	13	136	1 to 8
VR	8	329	1 to 9	3	62	1 to 7	12	141	1 to 9
VU	8	563	1 to 10	0	142	1 to 8	6	257	1 to 9
VV	75	259	1 to 10	21	37	1 to 5	87	72	1 to 8
AT	333	96	1 to 3	35	10	1 to 2	119	28	1 to 3
SL	145	245	1 to 7	47	36	1 to 3	91	70	1 to 7

The frequency range “Freq range” column describes the number of times a gene was present in different synteny blocks among species. Because the synteny blocks were calculated pairwise, and the same gene can be present multiple times, these values give a frequency reference. The RLK genes were split into two RLK and RLK-nonRD classes. ^a^Gene numbers reported are non-redundant; however, a gene can be present in one or more synteny blocks

The RLK/RLP gene frequency range described the number of times a gene could be present in different synteny blocks based on the pairwise comparison (Table 1). The RLK and RLP frequency range of genes in pairwise synteny blocks in the species comparison showed values between 1 and 13, with the exception of AT, which showed a low frequency range (1 to 3) in both plasma membrane classes. Interestingly, the legume RLK-nonRD proteins were located in syntenic blocks at a higher frequency (approximately 1 to 9) than for non-legumes proteins (approximately 1 to 5) (Table 1).

### Species synteny analysis

The identification of interspecies synteny blocks was calculated using the pairwise MCScanX approach to identify RLK and RLP syntenic blocks. The RLK/RLP proteins previously predicted [48] were used as a reference to select the species-specific syntenic blocks. The subset of synteny blocks of each species containing the plasma membrane receptors as a target did not automatically imply RLK or RLP transitivity, or a transitive relation among the synteny blocks; at the same time, the presence of an RLK/RLP in one of the species did not automatically imply their presence in the other species. Different criteria were used to split the legume/non-legume synteny block comparison to give an overview of the results. Also, all sets included VV because the divergence of grape occurred early in eudicot evolution and allows the split among Papilionoid species to be estimated [15]. The four sets were: 1) PV, GM: because PV is considered a diploid model for GM [55]; 2) MT, CC: because MT is considered a cool-season legume model [56] compared with CC, which is considered an orphan legume crop [7]; 3) VR, VU, VA: because this can be used as a reference to compare the legume *Vigna* genus; and 4) SL, AT, VV: because these sets correspond to the non-legumes species included and were a reference subset to compare distribution, conservation, and divergence among the outgroups.

### RLK among species synteny blocks

Among the 10-species evaluated, a total of 3049 pairwise RLK-RD alignments were observed. The blocks were split by the presence of RLK, but could also have RLK-nonRD and/or RLP present. The pairwise ratios of RLK genes present in synteny blocks among species were: 843 GM to 496 PV, 258 GM to 157 VV, and 91 PV to 60 VV. Among the GM and VU legumes, a 2:1 gene ratio of RLK/RLP was found in synteny blocks, and the plasma membrane receptors were distributed in multiple regions among all chromosomes. In the GM and PV comparison to VV, a decrease (70% or less) of RLK/RLP genes in synteny blocks was reported, and the VV-Chr 5 did not share any RLK/RLP synteny blocks (Additional file 11: Figure S4:A). The pairwise gene ratios of RLK among the MT and CC were: 93 MT to 64 CC, 46 MT to 31 VV, and 19 CC to 21 VV. The MT and CC legumes had approximately a 1:1 gene ratio of RLK/RLP shared in blocks and shared synteny fragments among almost all chromosomes, with the exception of CC-Chr5. The outgroup did not show shared synteny blocks with CC-Chr3, 5, 9, and 15 (Additional file 11: Figure S4:B).

For the pairwise gene ratio of RLK evaluated in synteny blocks among the *Vigna* genus (Additional file 11: Figure S4:C), the identified plasma membrane receptors present in synteny blocks were: 292 VR to 257 VA, 324 VR to 438 VU, 43 VR to 39 VV, 430 VA to 490 VU, 35 VA to 51 VV, and 86 VU to 98 VV. The legumes in this comparison set followed a 1:1 pairwise gene ratio, and almost all RLK/RLP genes (Table 1) were in syntenic and distributed fragments among all chromosomes. The pairwise ratio comparison of legumes against the outgroup show about a 90% reduction in RLK/RLP synteny. VU, VR, and VA do not share synteny blocks with VV-Chr 2, 3, 12, and 15. In contrast, the non-legume pairwise gene ratio shows 24 SL to 17 AT, 195 SL to 221 VV, and 46 AT to 84 VV (Additional file 11: Figure S4:D). The number of SL and VV RLKs syntenic blocks was proportionally higher compared with the other species. All chromosomes for the non-legumes were reported to have RLK synteny blocks.

### RLK-nonRD among legume/non-legume synteny blocks

Among the 10-species evaluated, a total of 715 alignments had the presence of RLK-nonRD. The predicted RLK-nonRD was used as a reference to target the synteny blocks. The alignments were not exclusive for the plasma membrane class and could also have the presence of RLK and/or RLP. The number of RLK-nonRD genes in a pairwise ratio among the synteny blocks was: 114 GM to 82 PV, 14 GM to 17 VV, and 5 PV to 17 VV. Among the GM and PV legumes, the RLK-nonRD ratio was 1:1, and all chromosomes had RLK/RLP genes present in syntenic blocks. In the legume/non-legume comparison, the proportion of syntenic RLK-nonRD genes was very low; also, 8 out of 19 chromosomes did not share synteny (Additional file 12: Figure S5:A). The pairwise gene ratio comparisons among MT and CC and the non-legume VV were: 18 MT to 9 CC, 2 CC to 3 VV, and 5 MT to 4 VV. In relation to the other legumes, MT and CC showed the lowest number of RLK-nonRD genes in synteny and, technically, only six blocks were shared with the VV non-legume species (5 of 19 VV-Chr involved) (Additional file 12: Figure S5:B).

The RLK-nonRD pairwise gene ratios identified among the *Vigna* genus were: 44 VR to 40 VA, 56 VR to 94 VU, 1 VR to 0 VV, 68 VA to 11 VU, 4 VA to 2 VV, and 10 VU to 7 VV. The *Vigna* species showed a synteny distribution of RLK-nonRD among all the chromosomes, and only eight synteny blocks were shared with the non-legume VV; 11 out of 19 VV-Chr did not share synteny (Additional file 12: Figure S5:C). The non-legumes showed pairwise gene ratios of 4 SL to 2 AT, 24 SL to 24 VV, and 3 AT to 6 VV (Additional file 12: Figure S5:D). As with the RLK, the proportion of RLK-nonRD shared between SL and VV was higher compared with the other species evaluated in this study. Not all non-legumes reported RLK-nonRD synteny in all chromosomes.

### RLP among synteny blocks

Among the 10-species evaluated, a total of 1361 alignments had the presence of RLP. The predicted RLP set was used as a reference to target the synteny blocks. The alignments were not exclusive for this plasma membrane class and could also contain other RLK and/or RLP. The pairwise ratios of RLP genes identified among the synteny blocks were: 252 GM to 159 PV, 57 GM to 6 VV, and 11 PV to 1 VV (Additional file 13: Figure S6:A). The RLP distribution among the GM and PV legumes involved fragments in all chromosomes. Like in the RLK ratio, the RLP had approximately a 2:1 ratio. The legume/non-legume ratio for RLP genes present in syntenic blocks was low; only seven VV genes were in synteny compared with 57 GM and 11 PV genes. In total, four VV-chromosomes were not in synteny with any of the legume species (Additional file 13: Figure S6). The pairwise ratio comparisons of RLP genes between the MT and the CC legumes were: 16 MT to 12 CC, 15 MT to 1 VV, and 5 CC to 1 VV. Among the MT and CC legumes, not all chromosomes shared RLP synteny blocks, and, compared with the non-legume species, 10 out of 19 VV-Chr did not share synteny (Additional file 13: Figure S6:B).

The *Vigna* genus reported pairwise ratios of RLP genes in synteny of: 14 VR genes to 105 VA genes, 13 VR genes to 42 VU genes, 1 VR gene to 1 VV gene, 120 VA genes to 28 VU genes, 6 VA genes to 0 VV genes, and 0 VU genes to 4 VV genes. Once again, as with RLK, all *Vigna* chromosomes shared fragments of synteny with RLP, whereas with the non-legumes, nine out of 19 VV-chromosomes did not display synteny (Additional file 13: Figure S6:C). The non-legumes presented showed RLP pairwise ratios of: 7 SL genes to 2 AT genes, 53 SL genes to 6 VV genes, and 14 AT genes to 2 VV genes (Additional file 13: Figure S6:D). With the exception of three out of 36 chromosomes in total (SL-Chr 12 and VV-Chr 9 and 13), synteny fragments occurred among all non-legume species (Additional file 13: Figure S6:D).

### *P. vulgaris* RLK and RLP synteny blocks as a model to compare the legume and non-legume species

**Fig. 5:**
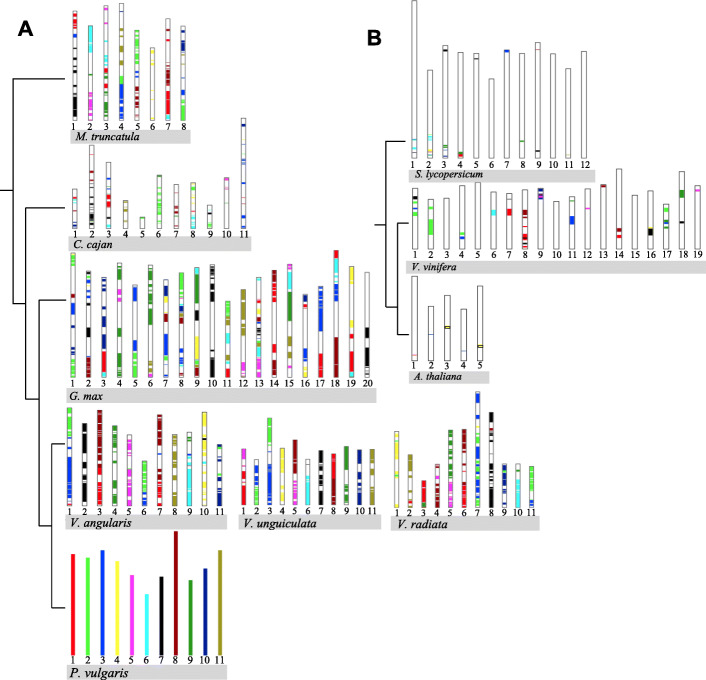
Distribution of *P. vulgaris* chromosome fragments with RLK/RLP. A. Legumes and B. non-legumes. The PV blocks include all RLK/RLP present in legumes and also all other proteins associated with the synteny blocks as a comparison reference. The 11 chromosomes of *P. vulgaris* are labeled with different colors and used as a structural genomic reference to shown the genomic fragments shared among the species with the presence of RLK/RLP

PV was used as a model to evaluate the RLK/RLP synteny block distribution among the legume/non-legume species. Syntenic blocks of the 11 PV chromosomes were distributed along all GM-Chrs. These PV blocks typically mapped to two GM blocks. PV-Chr7 was only present in GM-Chr10 and Chr20. Nine of 11 CC-Chrs had more than two PV-Chrs blocks, and PV-Chr11 and PV-Chr2 only shared multiple blocks with CC-Chr4 and CC-Chr5, respectively. Seven out of eight MT-Chrs had more than two synteny blocks from different PV-Chrs, and MT-Chr6 only had shared blocks that belonged to PV-Chr 4. PV-Chr7 and PV-Chr9 matched only with long fragments of VA-Chr2 and VA-Chr4, respectively. All VR-Chrs blocks shared between two to three synteny blocks with different PV-Chrs. The last comparison between PV and a *Vigna* species reported six out of 11 VU-Chrs sharing two long synteny blocks with PV-Chrs. The PV-Chr4, Chr7, Chr9, Chr10, and Chr11 showed a long fragment match with VU-Chr4, Chr7, Chr9, Chr10, and Chr11, respectively. The PV:VU chromosome distribution was notably similar. Also, the chromosome fragment evaluation between PV and the non-legumes identified nine out of 12 SL-Chrs shared small synteny regions with 10 PV-Chrs, and only PV-Chr5 did not share synteny with a SL chromosome. Also, five out of five AT-Chrs shared small regions with PV-Chr1, Chr3, and Chr4. Finally, 15 out of 19 VV-Chrs shared small synteny fragments with PV-Chrs regions (Fig. 5).

### *V. vinifera* RLK and RLP synteny blocks as a model to compare the legume and non-legume species

**Fig. 6:**
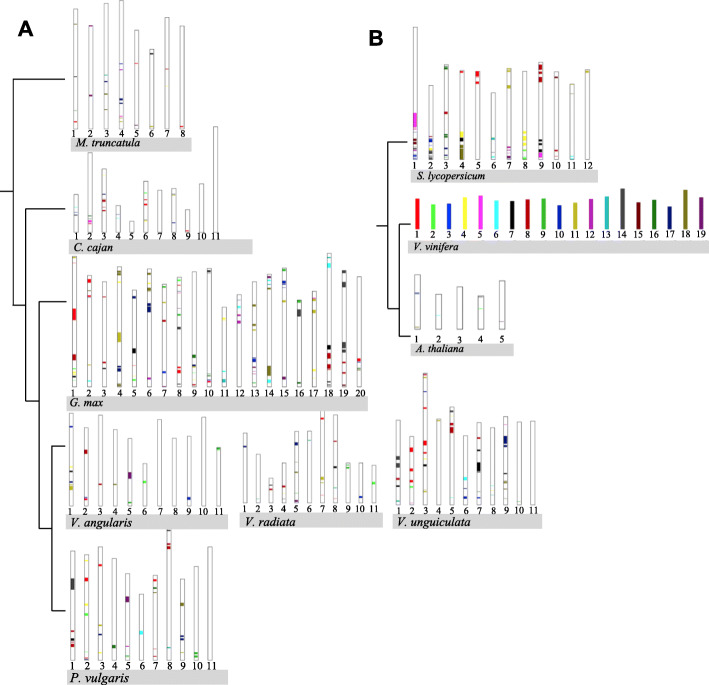
Distribution of *V. vinifera* chromosome fragments with RLK/RLP. A. Legumes and B. non-legumes. The PV blocks include all RLK/RLP present in legumes and also all other proteins associated with the synteny blocks as a comparison reference. The 19 chromosomes of *V. vinifera* are labeled with different colors and used as a structural genomic reference to shown the genomic fragments shared among the species with the presence of RLK/RLP

VV was also used as a model to evaluate the RLK/RLP synteny block distribution among the legume/non-legume species. Among the 10 species, VV shared more synteny blocks with the GM and the non-legume SL. Fragments of the 19 VV chromosomes were distributed along the 20 GM-Chrs sharing more than two VV-Chrs fragments. 10 out of 12 SL-Chrs share two or more VV-Chr fragments. Only 7 VV-Chrs fragments are shared with AT-Chrs, in fact, this is the species that shared fewer synteny blocks among the 10-species compared (Fig. 6).

### Identification of resistance RLK and RLP genes among legume/non-legume

**Fig. 7:**
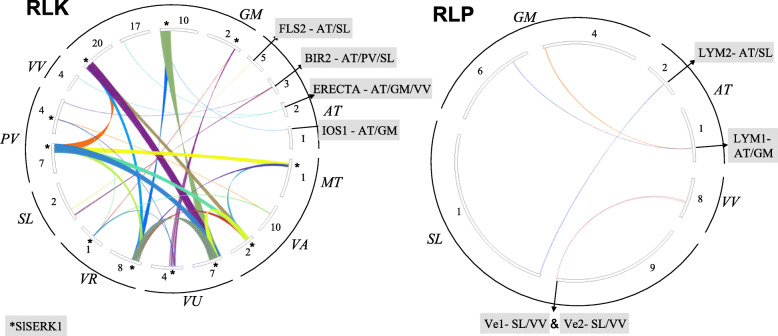
Synteny blocks with resistance RLK/RLP proteins among legumes/non-legumes. *Blocks that showed the presence of a protein with more than 90% identity to SlSERK1 (*S. lycopersicum*). Different copies of the gene were present in different species sharing the synteny blocks, but no blocks were shared with *S. lycopersicum*. *G. max* GM, *M. truncatula* MT, *V. angularis* VA, *V. unguiculata* VU, *V. radiata* VR, *S. lycopersicum* SL, *P. vulgaris* PV, *V. vinifera* VV, and *A. thaliana* AT

This part of the analysis assessed whether experimentally-validated RLK/RLP disease resistance genes (65 RLK and 28 RLP proteins reported, Additional file 14: Table S8) [48], were present in syntenic blocks among the legumes and non-legume species. The results of the pairwise comparisons indicated that the presence of the resistance plasma membrane in one species in a synteny block did not necessarily implicate the presence of the same experimentally validated RLK/RLP in the other species. Still, the synteny block must have had at least one RLK/RLP, and due to the required presence of at least five genes in common, the syntenic block was a valuable indicator of conserved synteny (Fig. 7).

Among the RLK proteins experimentally validated in *A. thaliana*, the FLS2 gene was present in a pairwise synteny block with *S. lycopersicum*; the BIR1 gene in a synteny block with *P. vulgaris* and *S. lycopersicum*; the ERECTA gene in a synteny block shared with *G. max* and *V. vinifera*; and the IOS1 gene in a synteny block shared with *G. max* (Fig. 7: RLK)*.* For *S. lycopersicum*, the SLSERK1 gene or a highly identical (< 90%) set of genes was present in shared synteny blocks among GM, PV, VR, VU, and VA; interestingly, the block was not shared with SL (Fig. 7, *SLSERK label shows the presence of the that gene among different chromosomes in the species evaluated). Further, for the RLP experimentally validated in *A. thaliana*, the LYM2 gene was present in synteny blocks shared with *S. lycopersicum*, and the LYM1 gene was in a shared synteny block with *G. max.* Finally, the RLP experimentally validated in *S. lycopersicum* and the target synteny blocks with the Ve1 and Ve2 genes were shared in a synteny block with *V. vinifera* (Fig. 7: RLP, and Additional file 15: Table S9.).

## Discussion

### Orthology analysis

The analysis revealed that almost all RLK and RLP orthologous genes belong to orthologous clusters rather than single-genes. This outcome suggests that WGD could contribute to the increased number of orthologous genes for RLK and RLP, corresponding to previous results reported for disease-resistant genes, or cytoplasmic R genes, in the legume family [17]. However, in order to create gene sets, single-copy gene families were identified in this study by counting the number of representatives of each species in a family. The process of identifying these families was complex due to issues with genome completeness and/or annotation, [57], and required high-quality genomic data to obtain reliable results, for that reason this study split the synteny analysis in two sets. Further, the proteins assigned to the orthologous and paralogous clusters could have been redundant due to the presence of protein isoforms.

In the evolution of higher eukaryotes, WGD followed by diploidization and the loss of many redundant gene duplicates, has been a recurrent process [58]. Due to recent duplications among legumes (Fig. 1), a high proportion of retained WGD genes in prior studies have been reported for the Papilionoids. With the extra WGD of *G. max*, a higher proportion of retained genes are present in this species compared to the other legume species. It was observed here that for RLK (Fig. 2, Figure S1) and RLP (Fig. 4, Figure S3) a high proportion of duplicated genes belong to multiple orthologous clusters compared with the singletons proteins. Interestingly, these lineage-specific duplications increase the diversity of protein families among lineages and are often important for stress? adaptation, especially for plants [23]. As such, the results reported here for the RLK and RLP represent part of this diversification process.

The analysis of plasma membrane receptor proteins results suggest different forces and mechanisms of the evolutionary process [59]. These forces and mechanisms are modulated by the evolutionary rate of gene duplication between orthologs that have paralogs (duplicates) evolving significantly slower than singletons [60]. Further, duplicate and singleton genes have significantly different sequence properties, expression patterns, molecular functions, and biological roles [61]. The expansion of the RLK gene-family in plants was hypothesized to have accelerated the evolution of proteins implicated in signal reception, particularly with the extra- or intracellular LRR domain. Under this expansion, the gene-family represents a plant-specific adaptation that leads to the production of numerous and variable cell surface and cytoplasmic receptors [62]. For example, FLS2, FLS3, XPS1, EFR, and Xa21, all members of the RLK-LRR-XII sub-family, have undergone significant gene expansion [63]. However, given that the receptor configuration must arise from a fusion between an RLP and RLCK, it is plausible that these RLK with innate immunity functions were originally RLP and RLCK that later fused together [64].

Over time, RLK and RLP have been exposed to a complex evolutionary process due to gene duplication and loss in plants [23]. The orthologous clustering process allowed eight single-copy gene clusters shared by MT, VU, PV, CC, GM and VV to be identified. Notably, the single-copy genes, typically involved in essential housekeeping functions, did not comprise a random segment of the genome, but rather their position was highly conserved across plant species [65]. Particularly, such single-copy genes have been recognized as molecular markers for inferring relationships of unresolved lineages [66]. In fact, recent research reported an optimal resolution of seed plant phylogeny, but required more than 100 single-copy genes [67].

### Synteny analysis

In evaluating the pairwise alignments calculated by MCScanX [68] to identify synteny blocks, about 1/3 of the alignments among the species showed the presence of RLK/RLP. Also, more than 75% RLK/RLP legumes genes are in synteny. The exception is MT where only ~ 50% of RLPs in that genome shared synteny with other species (Table 1). These results suggest that a high proportion of the plasma membrane receptors were conserved in legume syntenic blocks. Interestingly, the RLK/RLP not present in synteny blocks could be orthologs or singletons, In fact, according to the results reported for RLK/RLP proteins [48], 65% of RLK (Fig. 2) and 91% of RLP (Fig. 4) belonged to orthologous clusters, with the remaining genes classified as singletons. As expected, among legumes, not only were a higher number of orthologous proteins compared to non-legumes, but they were also present in synteny blocks. The proportion of plasma membrane receptors shared was lower among the AT, SL, and VV species.

Common bean is often considered a diploid relative of soybean, and its genome is considered a reference linking two duplicate soybean regions [69], and as expected the ratio of RLK/RLP present in synteny blocks among GM:PV was approximately 2:1 (Additional file 11: Figure S4:A, Additional file 12: Figure S5:A, and Additional file 13: Figure S6:A). This suggest that the ratio of the RLK/RLP present in the synteny blocks was also conserved in these plasma membrane receptors. Even though soybean has undergone a major duplication event [69], any sequence or sequence block unique to the soybean lineage will not have a common bean sequence signal, and any associated sequence duplication will not be uncovered [37]. This 2:1 ratio condition was identified among the RLK/RLP previously.

By comparison, among the the RLK/RLP ratio among MT:CC and CC:VV was 1:1, while MT:VV was 2:1 (Additional file 11: Figure S4:B, Additional file 12: Figure S5:B, and Additional file 13: Figure S6:B). The total number of RLK/RLP pairwise syntenic blocks shared between MT and CC, and between MT and VV, were the lowest among all pairwise comparisons (Additional file 5: Figure S4:B, Additional file 6: Figure S5:B, and Additional file 7: Figure S6:B). The 1:1 ratio of RLK/RLP was observed for all *Vigna* sp. comparisons. The RLK/RLP synteny blocks among the outgroup species showed higher syntenic block density among RLK compared with RLP. The 1:1 ratio was shared by SL:VV and AT:VV, and the SL:AT ratio was 2:1 (Additional file 11: Figure S4:D, Additional file 12: Figure S5:D, and Additional file 13: Figure S6:D). Even so, while the gene ratios among the species suggested a balanced relationship, the number of RLK/RLP genes shared among the legumes/non-legumes decreased among species with longer divergence times (Fig. 1). Regarding the synteny among the non-legume RLK/RLP genes, the AT gene frequency was lower compared with the results obtained for VV and SL.

### Synteny blocks using *P. vulgaris* and *V. vinifera* as structural genomic models

The evaluation of RLK/RLP fragment distribution in synteny blocks using the PV chromosomes as a reference species revealed diverse patterns of segmentation among species (Figs. 5 and 6), which is also evident in the synteny fragment comparison in the Fig. 5 using as a reference the *V. vinifera* genome. Particularly, with PV and GM, the RLK/RLP synteny block distribution was similar to the shared chromosome fragment distribution reported by previously [37]. Also, VU had a higher conservation with the common bean than with the other legume species. Most of the chromosomes between the adzuki bean (VA) and the common bean aligned in a way similar to that reported previously [10]. This corresponded with other studies where the adzuki bean (VA) species was shown to have a highly similar relationship to the common bean compared to soybean, pigeonpea, *Medicago*, chickpea, and lotus [10]. The PV and VU chromosomes showed the highest collinearity, based on RLK/RLK proteins, among the legumes studied here. These results showed collinearity of gene family members was maintained in the same manner as for the full genome sequence. Overall, these results suggest that *P. vulgaris* is an ideal anchor species for legume comparative genomics.

### Identification of resistance RLK/RLP genes among legume/non-legume synteny blocks

Because syntenic genes are orthologs, they are often considered to share similar functions [69]. The synteny blocks reported in Fig. 7 showed the experimentally validated resistance proteins for five RLK and four RLP shared among different pairwise blocks. This result suggests that further analysis must be applied to functionally evaluate the genes/proteins present in those pairwise blocks, and that they do not necessarily show the presence of the same resistance RLK/RLP proteins. The presence of the other proteins that belong to the blocks could relate to the functional association to resistance, but this hypothesis also needs to be confirmed. Even so, the syntenic blocks could be used as a reference to build a targeted co-expression network and infer probable functional interactions among the genes based on the RLK/RLP proteins.

## Conclusions

The dynamic evolution of RLK and RLP in the legume family is evidence of a complex history of gene duplication and loss in relation to WGD events. Regarding gene-family expansion, the LRR-RLK/RLP proteins comprised more than 60% of the plasma membrane legume receptors evaluated. The seven-legume species shared more RLK/RLP genes among synteny blocks compared with AT, SL, and VV, suggesting patterns of evolutionary conservation among these species relative to the non-legumes. The comparative syntenic analysis of the RLK and RLP genes [48] was an important computational annotation strategy that revealed that plasma membrane receptors were distributed and shared in syntenic blocks among dicots and between legume and non-legume species that most likely predated the evolutionary appearance of these different lineages. *M. truncatula* had the fewest shared syntenic blocks with the other legumes, which probably reflects that it is the only Galeogoid legume whereas the other legume species are members of the Phaseolids. For the synteny blocks shared among legumes/non-legumes, AT shared the lowest number of RLK/RLP genes with the legumes (Table 1). The GM:PV 2:1 ratio of RLK/RLP among the synteny blocks also suggests that these types of plasma receptors typically follow this ratio indicative of the WGD of soybean, which has been previously reported among these legumes [37]. The *Vigna* genus shared long fragments of chromosomes with RLK/RLP in synteny with PV. Further, *P. vulgaris* and *V. unguiculata* displayed the most similar RLK/RLP chromosome fragment distribution among all legume/non-legume comparisons, a result consistent with their high collinearity [46]. Significantly, all RLK-nonRD genes present in the PV and VU were in synteny blocks, suggesting a highly conserved relationship among this type of RLK between these species. This could also be related to the fact that that these two species have the most complete reference genome sequences. Further analysis is required to confirm that RLK/RLP synteny blocks with the experimentally validated RLK/RLP resistance genes can be used to discover functionally relate candidate resistance factors in legumes. Overall, it was again confirmed that PV could be used as an anchor species for comparative legume genomics [55].

## Methods

### Datasets

The orthology analysis involved the RLK-RD (Additional file 16: Table S10), RLP (Additional file 17: Table S11), and RLK-nonRD (Additional file 18: Table S12) proteins classified previously for seven-legume species (soybean, common bean, barrel medic, mungbean, cowpea, adziki bean and pigeonpea) [48]. Grape (*V. vinifera*) (as the closest legume “outgroup”), tomato (*S. lycopersicum*), and *Arabidopsis* were included as plant models in dicots [48] . The RLK-nonRD dataset represented about 10% of the total RLK proteins, but it was extracted to evaluate its relationship since it is potentially associated with innate immune receptors that recognize conserved microbial signatures [70]. The experimentally-validated RLK and RLP were also included in the evaluations. Proteins with the presence of a string region with more than four undefined amino acids, labeled as “X” in a continuous position in 50% or more of its whole sequence, were excluded.

The homology inference to RLK, RLK-nonRD, and RLP proteins among all species was calculated with the OrthoMCL [71] tool that reports orthologous clusters, using a Blastp threshold of E-value 1e − 5, and a MCL inflation parameter of 1.5 (default parameters). The results were visualized with OrthoVenn [21]. The criteria to be included in the orthology analysis followed this order based on the genomes evaluated: A) among the legumes selected, included one species per genera, B) prioritized the genome species included from the Phytozome repository due to its quality standards [72], and C) included the closest outgroup. The orthology analysis for *V. radiata*, *V. angularis*, *A. thaliana*, and *S. lycopersicum* was reported as supplementary data. This process allowed the identification of putative functional orthologous clusters (orthologous and paralogous), single-copy gene clusters, and singletons.

Different datasets were used and adjusted for the synteny analysis. The whole protein dataset and the gene annotation for each genome were collected. That dataset was used as an input to build a blast database using only the genes located on pseudochromosomes. The genes present in the chloroplast chromosome (ChrC), mitochondria chromosome (ChrM), and scaffolds were excluded. Two genomic databases were used to obtain the legume/non-legume genomes: the NCBI database (https://www.ncbi.nlm.nih.gov/) for three species and the Phytozome repository database (https://phytozome.jgi.doe.gov/pz/portal.html) for the other seven species (Table 2).

**Table 2:** Summary of genomes

Species	Database	File name	N. of genes	N. of proteins	N. of chr
*V. radiata*	NCBI	GCF_000741045.1_Vradiata_ver6	34,911	35,143	11
*C. cajan*	NCBI	GCA_000340665.1_C.cajan_V1.0	23,374	48,331	11
*V. angularis*	NCBI	annotation release 100	22,276	37,769	11
*G. max*	Phytozome	gmax_275_wm82.a2.v1	55,589	88,647	20
*M. truncatula*	Phytozome	Mtruncatula_285_Mt4.0v1	48,338	62,319	8
*P. vulgaris*	Phytozome	Pvulgaris_442_v2.1	27,012	36,995	11
*V. unguiculata*	Phytozome	Vunguiculata_469_v1.1	28,881	42,287	11
*A. thaliana*	Phytozome	Athaliana_167_TAIR10	27,206	35,386	5
*S. lycopersicum*	Phytozome	Slycopersicum_390_ITAG2.4	33,838	34,725	12
*V. vinifera*	Phytozome	Vvinifera_145_Genoscope.12X	23,647	26,346	19

Syntenic block discovery focused on predicted RLK and RLP proteins (Additional file 16: Table S10, Additional file 17: Table S11, and Additional file 18: Table S12 [48]. The target blocks were used to evaluate synteny of RLK/RLP containing blocks in legume/non-legume species following a comparative genomic approach, which also allowed patterns of conservation and/or divergence among and between them to be identified. At the same time, pathogen resistance RLK/RLP proteins were also used as a reference to track the presence of syntenic blocks containing experimentally-validated RLK and RLP among legumes/non-legumes. This approach does not necessarily imply the presence of an experimentally-validated RLK/RLP protein [48] must be shared among species, but if the interspecies synteny blocks were shared, the match was reported.

### Interspecies identification of synteny blocks

The database and the blastp for the calculation of the synteny block input were built using a ncbi-blast-2.7.1+ package (makeblastdb and blastp). The query for the makeblastdb script was the whole set of proteins reported for the legume/non-legume species. The parameters for the blastp were blastp -outfmt 6 -evalue 1e-10 -max_target_seqs 5. The output obtained from the blast process and the GFF annotation of the 10 species (seven legumes/three non-legumes) were used as the input for the synteny blocks calculation. The interspecies syntenic blocks were calculated using the MCScanX tool [68] with the following parameters: match-score, final score = match_score + num_gaps * gap_penalty (default: 50); gap-penalty, gap penalty (default: − 1); match-size, the number of genes required to call a collinear block (default: 5); E-value, alignment significance 1e-10; max-gaps, maximum gaps allowed (default: 25); and overlap-window, maximum distance 10,000 (number of nucleotides among genes) to collapse blast matches (default: 5) and the patterns of collinear blocks: 1 inter-species. The approach identified two or more species shared a pairwise synteny block that had at least five genes shared with an E-value <1e-10 in a maximum range of 10,000 nucleotides. Also, the script dissect_multiple_alignment was used to subset all results and obtain a reference among the species compared. For the figures, the MCScanX package circle_plotter and bar_plotter were employed. After the set of synteny blocks were identified, in-house scripts were developed to subset the MCScanX collinearity output file, isolating the synteny blocks with RLK/RLP.

With the goal of identifying synteny blocks among the species with identical and/or highly identical resistance RLK/RLP blocks, an identity clustering analysis was applied. The analysis compared the predicted RLK/RLP reported (Additional file 9: Table S3 and Additional file 10: Table S4) against the experimentally-validated resistance RLK/RLP (Additional file 11: Table S5) using the CD-HIT [73] tool. Specifically, the script “cd-hit-2d” with the parameters -c 0.9 -n 5 was applied. Approximately 75% of the experimentally validated resistance RLK/RLP were encoded by *A. thaliana* or *S. lycopersicum* genes. This approach allowed the proteins that share more than 90% of their identity to be determined. The identical and highly identical resistance RLK/RLP proteins were used to identify synteny blocks in the non-legume species *Arabidopsis* and *S. lycopersicum* that were shared among the legumes. In-house scripts (https://github.com/drestmont/plant_rlk_rlp/) were used to identify the presence of resistance genes among the synteny blocks. The whole process required 10 servers (each one with 16 cores and 32 GB ram) running in parallel for 2 weeks. BLASTP used 95% of the computational time. The process was run at the scientific cluster at Universidad Nacional de Colombia.

## Supplementary Information

**Additional file 1: Table S1**. Orthologous, paralogus and single-copy gene clusters of RLK among VV, CC, PV, VU, MT, and GM.

**Additional file 2: Figure S1.** Summary of the RLK orthology analysis among VR, VA, AT, SL, and VV. A. Venn diagram showing the distribution of shared gene families (orthologous clusters) among VR, VA, AT, SL, and VV. B1. The numbers refer to all the clusters in the species, including orthologs and in-paralogs. B2. Distribution of the number of species present in ortholog clusters, one or share elements among species. C. Summary of the total number of proteins, clusters, and singletons within each species. The RLK and its isoforms and nonRD proteins were included in this figure. 28 single–copy gene clusters were reported among the species evaluated.

**Additional file 3: Table S2**. orthologous, paralogous and single-copy gene clusters of RLK-RD among VV, AT, SL, VR, and VA.

**Additional file 4: Table S3**. orthologus, paralogus and single-copy gene clusters of RLK-nonRD among VV, CC, PV, VU, MT, and GM.

**Additional file 5: Figure S2**. Summary of the RLK-nonRD orthology analysis among VR, VA, AT, SL, and VV. A. Venn diagram showing the distribution of shared gene families (orthologous clusters) among VR, VA, AT, SL, and VV. B1. The numbers refer to all the clusters in the species, including orthologs and in-paralogs. B2. Distribution of the number of species present in orthologs clusters, one or share elements among species. C. Summary of the total number of proteins, clusters, and singletons within each species. The RLK and its isoforms and nonRD proteins were included in this Fig. 3 single-copy gene clusters were reported among the species evaluated.

**Additional file 6: Table S4.** Orthologous, paralogous and single-copy gene clusters of RLK-nonRD among VV, AT, SL, VR, and VA.

**Additional file 7: Table S5**. orthologus, paralogus and single-copy gene clusters of RLP among VV, CC, PV, VU, MT, and GM.

**Additional file 8: Figure S3**. Summary of the RLP orthology analysis among VR, VA, AT, SL, and VV. A. Venn diagram showing the distribution of shared gene families (orthologous clusters) among VR, VA, AT, SL, and VV. B1. The numbers refer to all the clusters in the species, including orthologs and in-paralogs. B2. Distribution of the number of species present in orthologs clusters, one or share elements among species. C. Summary of the total number of proteins, clusters, and singletons within each species. The RLK and its isoforms and nonRD proteins were included in this evaluation report. 4 single-copy gene clusters were reported among the species evaluated.

**Additional file 9: Table S6**. Orthologous, paralogous and single-copy gene clusters of RLP among VV, AT, SL, VR, and VA.

**Additional file 10: Table S7**. Summary of the total genes evaluated among legumes/non-legumes in the synteny analysis.

**Additional file 11: Figure S4.** Distribution of RLK present in synteny blocks. Chromosomes of the species evaluated. For visual purposes, the RLK identified in a synteny block were used as a reference to plot the circles. The RLK-nonRD were excluded in the figure VV was included in all figures as an outgroup for legumes and also to compare results among AT and SL. A) *G. max* GM, *P. vulgaris* PV, and *V. vinifera* VV. B) *M. truncatula* MT, *C. cajan* CC, and VV. C) *V. radiata* VR, *V. angularis* VA, *V. unguiculata* VU, and VV. D) *A. thaliana* AT, *S. lycopersicum* SL, and VV.

**Additional file 12: Figure S5.** Distribution of RLK-nonRD present in synteny blocks. Chromosomes of the species evaluated. For visual purposes, the RLK identified in a synteny block were used as a reference to plot the circles. VV was included in all figures as an outgroup for legumes and also to compare results among AT and SL. A) *G. max* GM, *P. vulgaris* PV, and *V. vinifera* VV. B) *M. truncatula* MT, *C. cajan* CC, and VV. C) *V. radiata* VR, *V. angularis* VA, *V. unguiculata* VU, and VV. D) *A. thaliana* AT, *S. lycopersicum* SL, and VV.

**Additional file 13: Figure S6.** Distribution of RLP present in synteny blocks. Chromosomes of the species evaluated. For visual purposes, the RLK identified in a synteny block were used as a reference to plot the circles. The RLK were excluded, and VV was included in all figures as an outgroup for legumes and also to compare results among AT and SL. A) *G. max* GM, *P. vulgaris* PV, and *V. vinifera* VV. B) *M. truncatula* MT, *C. cajan* CC, and VV. C) *V. radiata* VR, *V. angularis* VA, *V. unguiculata* VU, and VV. D) *A. thaliana* AT, *S. lycopersicum* SL, and VV.

**Additional file 14: Table S8.** Experimentally-validated RLK, RLP, and R gene proteins used to evaluate the prediction.

**Additional file 15: Table S9.** Synteny block identification of resistance RLK and RLP genes among legumes/non-legumes reported on Fig. 7.

**Additional file 16: Table S10.** Protein ids of the 10 species evaluated that are classified as RLK.

**Additional file 17: Table S11.** Protein ids of the 10 species evaluated that are classified as RLP.

**Additional file 18: Table S12.** RLK-nonRD IDs identified among the species evaluated.

## Data Availability

All data analyzed during this study is available in Phytozome, the NCBI and Pfam database. The datasets generated and/or analyzed during the current study are available in the github repository, https://github.com/drestmont/plant_rlk_rlp/

## Abbreviations

RLK: Receptor-Like Kinase (Syn: RLK-RD)
RLP: Receptor-Like Protein
RLCK: Receptor-Like Cytoplasmic Protein Kinase
RLK-nonRD: Receptor-Like Kinase with absence of an arginine (R) located before a catalytic aspartate (D)
GM: *Glycine max*
PV: *Phaseolus vulgaris*
MT: *Medicago truncatula*
VR: *Vigna radiata*
VU: *Vigna unguiculata*
VA: *Vigna angularis* var. *Angularis*
CC: *Cajanus cajan*
AT: *Arabidopsis thaliana*
SL: *Solanum lycopersicum*
VV: *Vitis vinifera*
