# Supplementary material for: Orthology and synteny analysis of receptor-like kinases “RLK” and receptor-like proteins “RLP” in legumes
Source: BMC Genomics. 2021 Feb 10;22:113. doi: 10.1186/s12864-021-07384-w (PMC7874474; doi:10.1186/s12864-021-07384-w)
Supplement: Supplementary file 5 — Additional file 5: Figure S2. Summary of the RLK-nonRD orthology analysis among VR, VA, AT, SL, and VV. A. Venn diagram showing the distribution of shared gene families (orthologous clusters) among VR, VA, AT, SL, and VV. B1. The numbers refer to all the clusters in the species, including orthologs and in-paralogs. B2. Distribution of the number of species present in orthologs clusters, one or share elements among species. C. Summary of the total number of proteins, clusters, and singletons within each species. The RLK and its isoforms and nonRD proteins were included in this Fig. 3 single-copy gene clusters were reported among the species evaluated. [file 12864_2021_7384_MOESM5_ESM.pptx]

## Slide 1
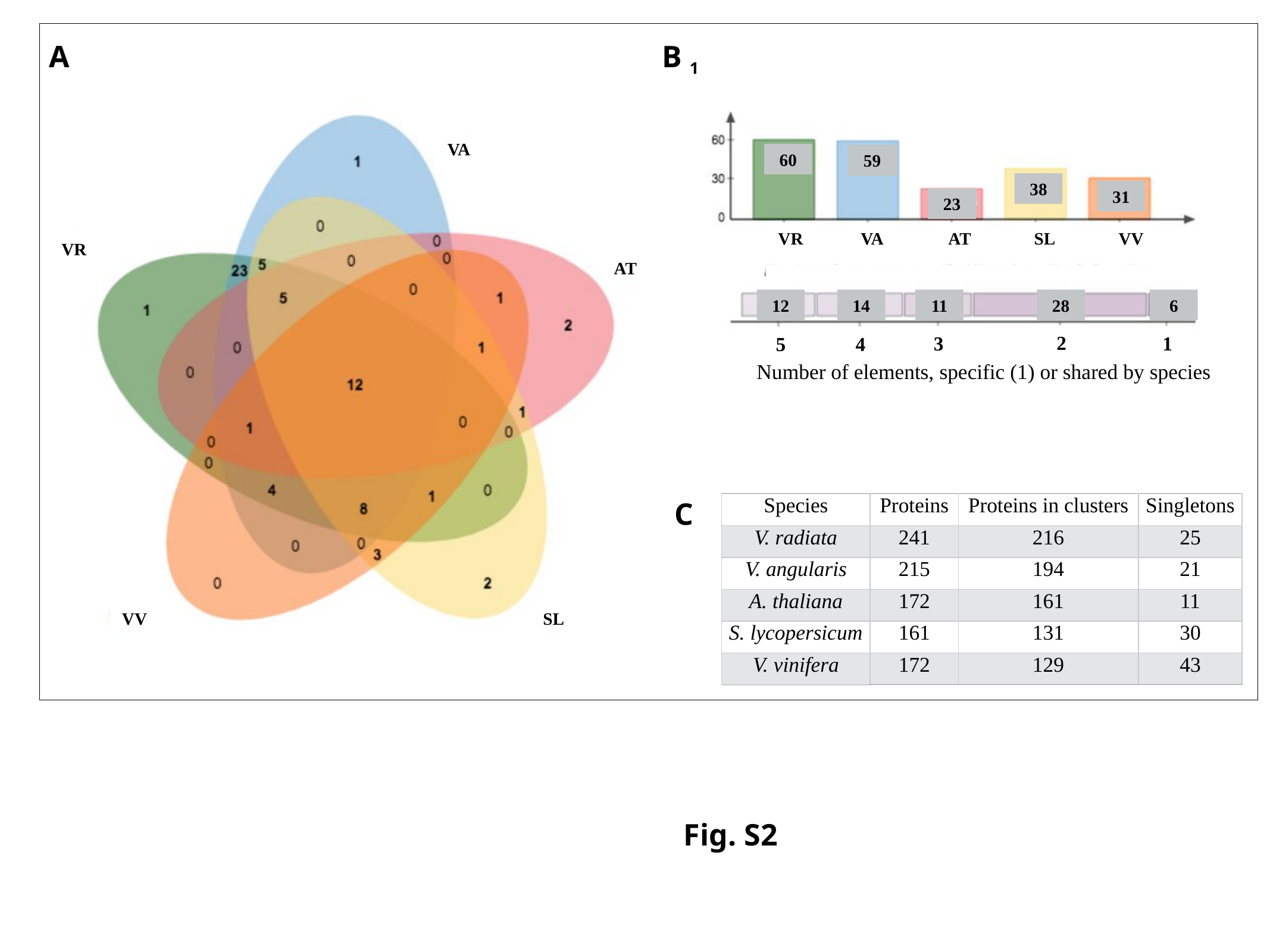

A
B
1
VA
60
59
38
31
23
VR
VA
AT
SL
VV
VR
AT
12
14
11
28
6
2
1
3
5
4
Number of elements, specific (1) or shared by species
C
| Species | Proteins | Proteins in clusters | Singletons |
| --- | --- | --- | --- |
| V. radiata | 241 | 216 | 25 |
| V. angularis | 215 | 194 | 21 |
| A. thaliana | 172 | 161 | 11 |
| S. lycopersicum | 161 | 131 | 30 |
| V. vinifera | 172 | 129 | 43 |
VV
SL
Fig. S2
