# Supplementary figures and images for: Orthology and synteny analysis of receptor-like kinases “RLK” and receptor-like proteins “RLP” in legumes
Source: BMC Genomics. 2021 Feb 10;22:113. doi: 10.1186/s12864-021-07384-w (PMC7874474; doi:10.1186/s12864-021-07384-w)

## Slide 1
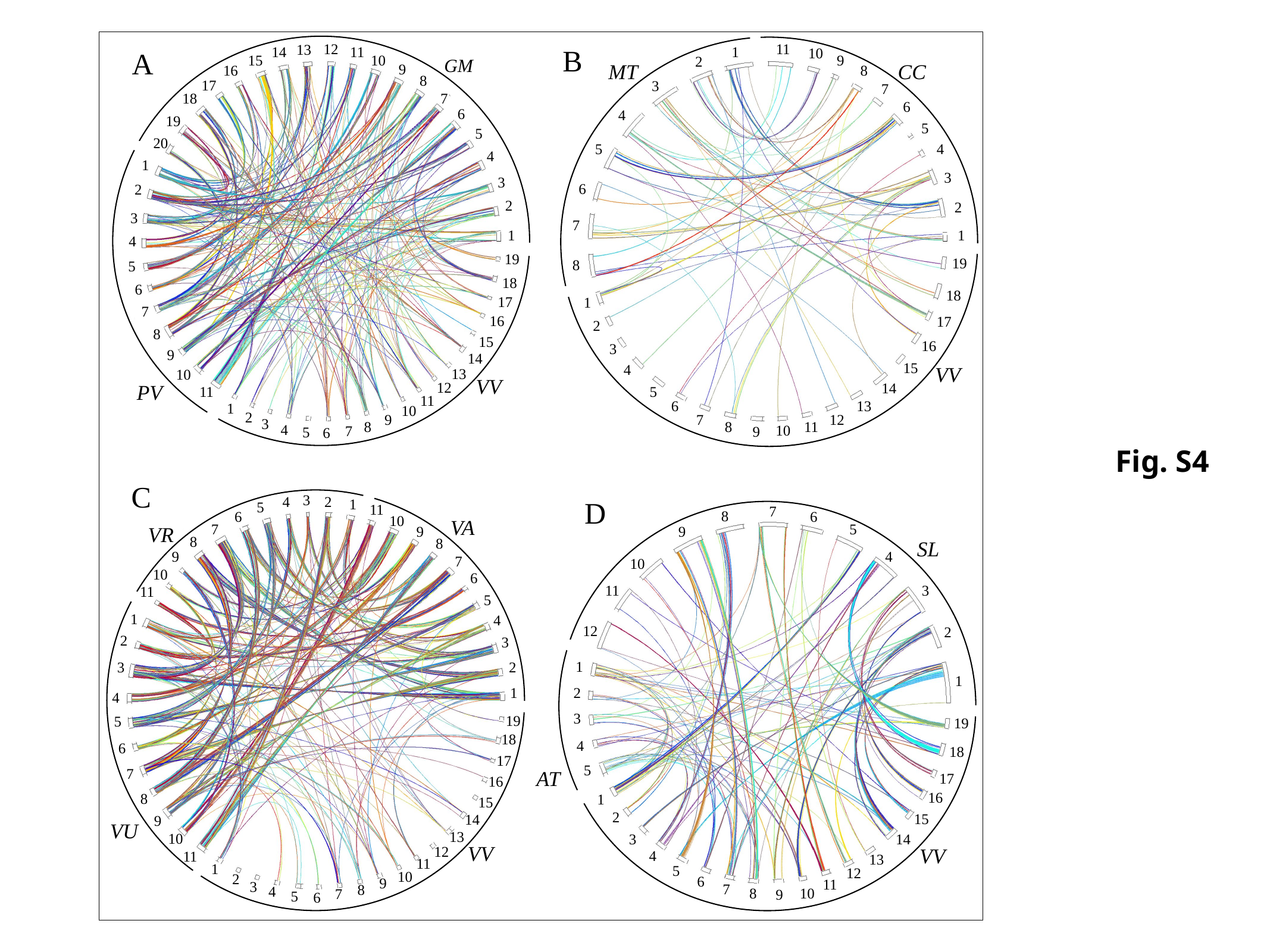

12
11
13
1
14
11
B
10
A
15
10
9
2
GM
MT
CC
9
16
8
8
17
3
7
18
7
6
6
4
19
5
5
20
5
4
4
1
3
3
6
2
2
2
3
7
1
1
4
19
19
8
5
18
6
18
17
1
7
16
17
2
8
15
16
3
9
14
15
4
VV
13
10
VV
12
14
PV
5
11
11
6
13
1
10
2
7
12
9
3
8
11
8
4
10
7
9
5
6
Fig. S4
C
3
4
2
1
D
5
11
7
8
6
6
10
VA
VRA
7
5
VR
9
9
8
8
SL
9
4
7
10
10
6
11
3
11
5
1
4
12
2
2
3
1
3
2
1
1
2
4
3
19
5
19
18
4
6
18
17
5
7
AT
17
16
16
8
1
15
2
15
14
9
VU
13
10
14
3
VV
12
VV
4
11
13
11
1
5
12
10
2
6
9
11
3
7
8
4
10
8
7
9
5
6
9

Supplement: Supplementary file 11 — Additional file 11: Figure S4. Distribution of RLK present in synteny blocks. Chromosomes of the species evaluated. For visual purposes, the RLK identified in a synteny block were used as a reference to plot the circles. The RLK-nonRD were excluded in the figure VV was included in all figures as an outgroup for legumes and also to compare results among AT and SL. A) G. max GM, P. vulgaris PV, and V. vinifera VV. B) M. truncatula MT, C. cajan CC, and VV. C) V. radiata VR, V. angularis VA, V. unguiculata VU, and VV. D) A. thaliana AT, S. lycopersicum SL, and VV. [file 12864_2021_7384_MOESM11_ESM.pptx]

## Slide 1
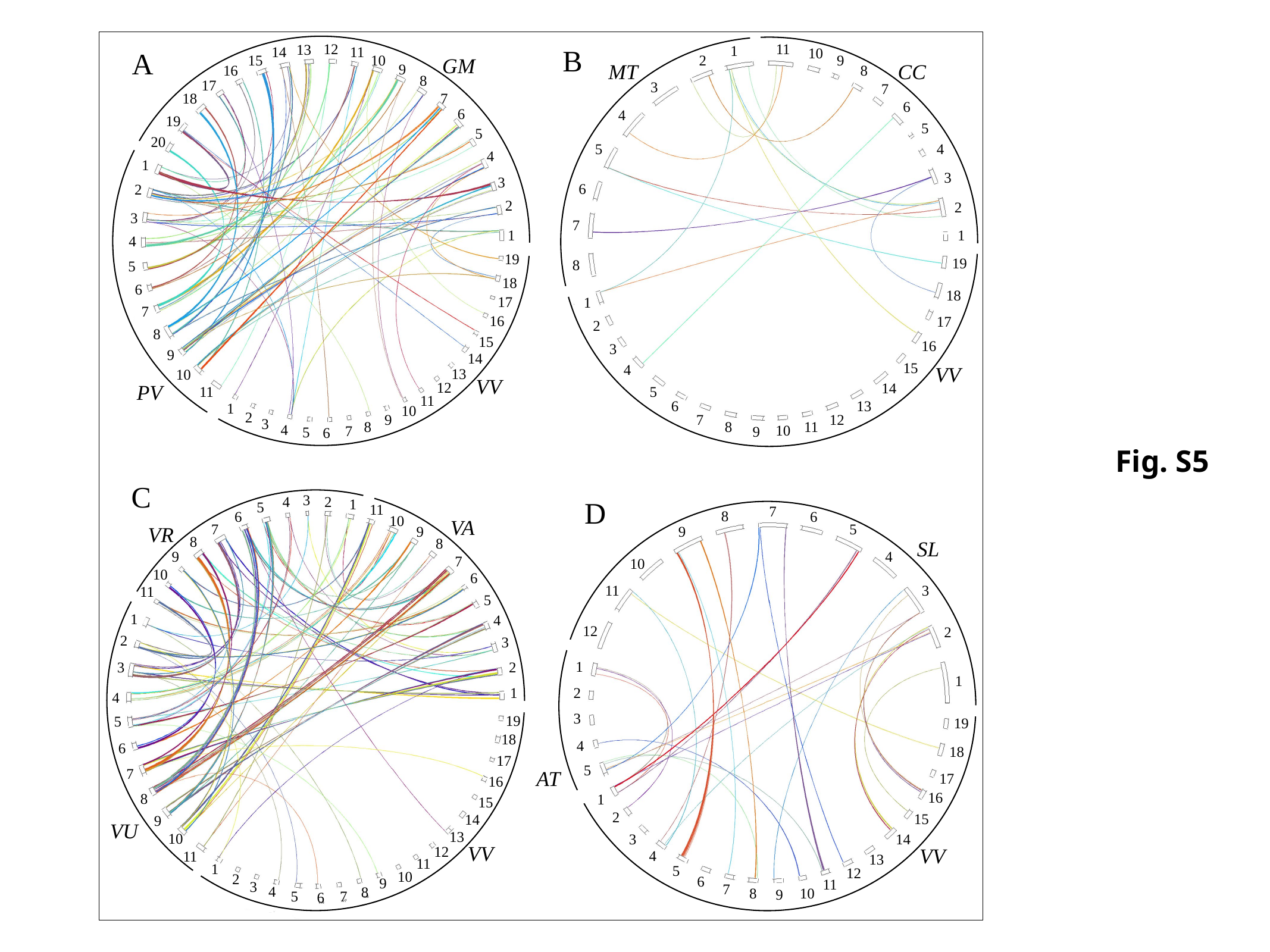

12
11
13
1
14
11
B
10
A
15
10
2
9
GM
MT
CC
9
16
8
8
17
3
7
18
7
6
6
4
19
5
5
20
5
4
4
1
3
3
6
2
2
2
3
7
1
1
4
19
19
8
5
18
6
18
17
1
7
16
17
2
8
15
16
3
9
14
15
4
VV
13
10
VV
12
14
PV
5
11
11
6
13
1
10
2
7
12
9
3
8
11
8
4
10
7
9
5
6
Fig. S5
C
3
4
2
1
D
5
11
7
8
6
6
10
VA
7
5
VR
9
9
8
8
SL
9
4
7
10
10
6
11
3
11
5
1
4
12
2
2
3
1
3
2
1
1
2
4
3
19
5
19
18
4
6
18
17
5
7
AT
17
16
16
8
1
15
2
15
14
9
VU
13
10
14
3
VV
12
VV
4
11
13
11
1
5
12
10
2
6
9
11
3
7
4
8
10
8
9
5
7
6

Supplement: Supplementary file 12 — Additional file 12: Figure S5. Distribution of RLK-nonRD present in synteny blocks. Chromosomes of the species evaluated. For visual purposes, the RLK identified in a synteny block were used as a reference to plot the circles. VV was included in all figures as an outgroup for legumes and also to compare results among AT and SL. A) G. max GM, P. vulgaris PV, and V. vinifera VV. B) M. truncatula MT, C. cajan CC, and VV. C) V. radiata VR, V. angularis VA, V. unguiculata VU, and VV. D) A. thaliana AT, S. lycopersicum SL, and VV. [file 12864_2021_7384_MOESM12_ESM.pptx]

## Slide 1
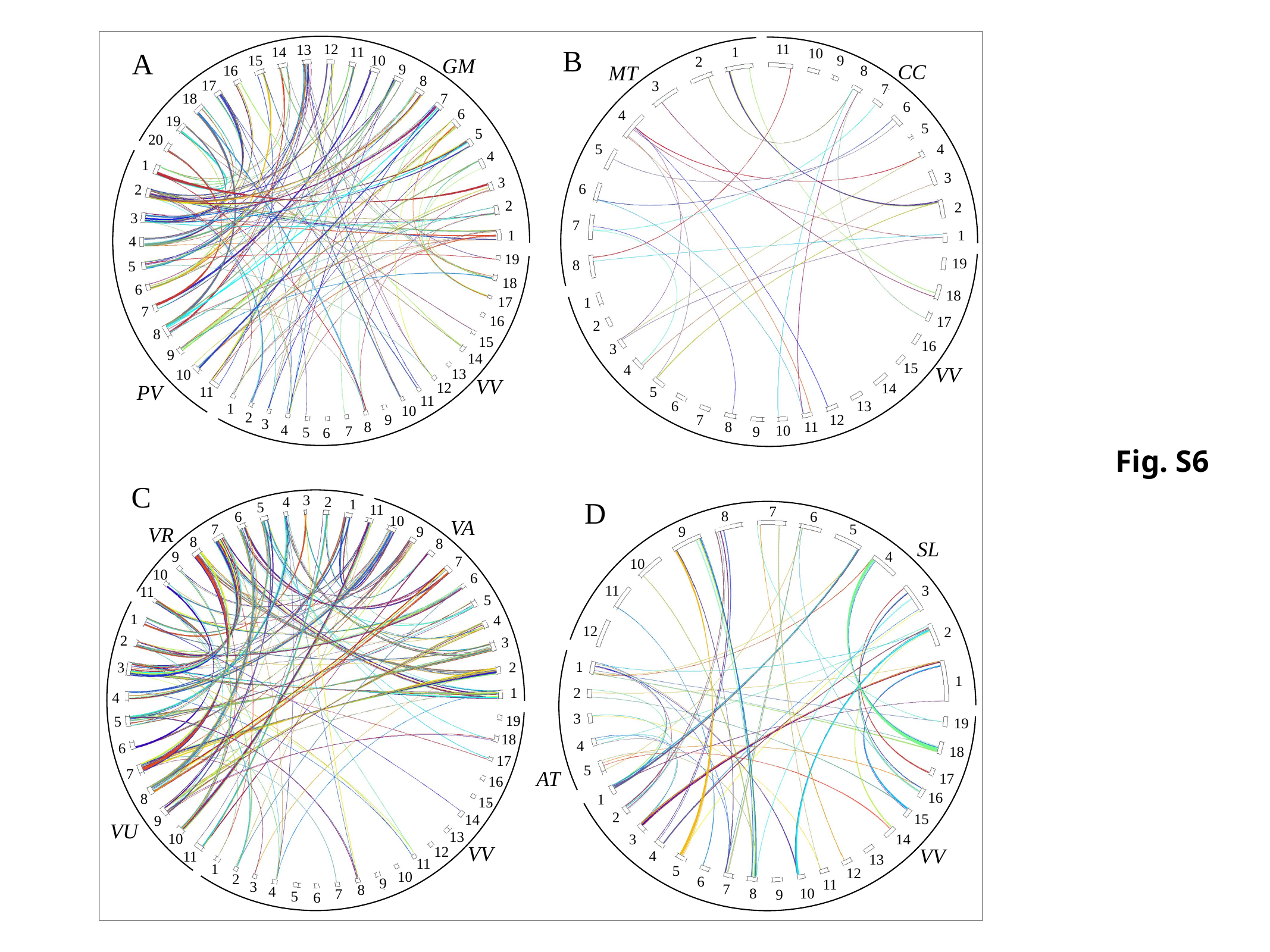

12
11
13
1
14
11
B
10
A
15
10
9
2
GM
CC
9
MT
16
8
8
17
3
7
18
7
6
6
4
19
5
5
20
5
4
4
1
3
3
6
2
2
2
3
7
1
1
4
19
19
8
5
18
6
18
17
1
7
16
17
2
8
15
16
3
9
14
15
4
VV
13
10
VV
12
14
PV
5
11
11
6
13
1
10
2
7
12
9
3
8
11
8
4
10
7
9
5
6
Fig. S6
C
3
4
2
1
D
5
11
7
8
6
6
10
VA
7
5
VR
9
9
8
8
SL
9
4
7
10
10
6
11
3
11
5
1
4
12
2
2
3
1
3
2
1
1
2
4
3
19
5
19
18
4
6
18
17
5
7
AT
17
16
16
8
1
15
2
15
14
9
VU
13
10
14
3
VV
12
VV
4
11
13
11
1
5
12
10
2
6
9
11
3
7
8
4
10
8
7
9
5
6

Supplement: Supplementary file 13 — Additional file 13: Figure S6. Distribution of RLP present in synteny blocks. Chromosomes of the species evaluated. For visual purposes, the RLK identified in a synteny block were used as a reference to plot the circles. The RLK were excluded, and VV was included in all figures as an outgroup for legumes and also to compare results among AT and SL. A) G. max GM, P. vulgaris PV, and V. vinifera VV. B) M. truncatula MT, C. cajan CC, and VV. C) V. radiata VR, V. angularis VA, V. unguiculata VU, and VV. D) A. thaliana AT, S. lycopersicum SL, and VV. [file 12864_2021_7384_MOESM13_ESM.pptx]
